# *Hyphoderma pinicola* sp. nov. of *H. setigerum* complex (Basidiomycota) from Yunnan, China

**DOI:** 10.1186/s40529-014-0071-5

**Published:** 2014-10-09

**Authors:** Eugene Yurchenko, Sheng-Hua Wu

**Affiliations:** 1 Department of Biotechnology, Paleski State University, Dnyaprouskai flatylii str. 23, Pinsk, BY-225710 Belarus; 2 Department of Biology, National Museum of Natural Science, Taichung, 404 Taiwan

**Keywords:** Corticioid fungi, Meruliaceae, Polyporales, Taxonomy

## Abstract

**Backgroud:**

*Hyphoderma setigerum* (Fr.) Donk is a white-rot wood-decaying corticoid fungal species. It occurs worldwide from tropical to temperate regions. However, taxonomic studies in recent decades showed that *H. setigerum* is a species complex with four separate species, before this study.

**Results:**

*Hyphoderma pinicola* sp. nov. was collected on dead wood of *Pinus yunnanensis* Franch. in the temperate montane belt at 2200–2400 m altitudes, in Yunnan Province of China. Within the *H. setigerum* complex this new taxon is distinguished by having 2-sterigmate basidia, long basidiospores, and nearly naked septocystidia. A description and illustrations of this new species are provided, along with a key to five species of the *H. setigerum* complex. Phylogenetic reconstruction based on 5.8S-ITS2 sequences indicated that *H. pinicola* belongs to the *H. setigerum* complex and has a separate position within the clade including *H. subsetigerum* and *H. setigerum* s.s. Bayesian inference of phylogeny based on two datasets, ITS and 28S nuclear ribosomal DNA sequences, confirmed the independent status of *H. pinicola*.

**Conclusion:**

Morphological and phylogenetic studies showed that *H. pinicola* represents a fifth species of *H. setigerum* complex.

**Electronic supplementary material:**

The online version of this article (doi:10.1186/s40529-014-0071-5) contains supplementary material, which is available to authorized users.

## Background

*Hyphoderma* Wallr. is the largest genus of Basidiomycota with resupinate non-poroid basidiomata. Currently, 103 species are recognized under *Hyphoderma* in *Index Fungorum* (Kirk, [2014]). According to Dai ([2011]), 24 species of *Hyphoderma* s.l. (including *Mutatoderma* (Parmasto) C.E. G mez and *Peniophorella* P. Karst.) were listed in the mycobiota of China. *Hyphoderma setigerum* (Fr.) Donk occurs worldwide from tropical to temperate regions. However, taxonomic studies in recent decades showed that *H. setigerum* is a species complex with four species. A new species belonging to the *H. setigerum* complex is described in the present paper. This new taxon is based on specimens collected in 2001 on dead branches of *Pinus yunnanensis* Franch., from the temperate montane belt of Yunnan Province, China.

## Methods

### Reference herbarium materials and study of the morphology

The specimens studied of this new species are deposited in the herbaria TNM and MSK (herbarium acronyms follow *Index Herbariorum*, http://sweetgum.nybg.org/ih). The isolate is kept in the culture collection of TNM.

Description of macromorphology is based on dry basidiomata. Microscopic measurements and drawings were made from material mounted in 3% KOH water solution. Melzer’s reagent was used to examine amyloidity or dextrinoidity of spore walls, but also to study crystalline incrustations on hyphae, or in the hymenium. Cyanophily of the spore wall was tested in cotton blue-lactophenol solution. To determine average spore size, 30 randomly selected spores from a squash basidioma preparation were measured.

### DNA extraction, amplification, and sequencing

Nuclear ribosomal DNA sequences of *Hyphoderma* were analyzed in addition to morphological study. The material for DNA isolation was mycelium grown in pure culture (*Wu 0108–36*), and basidioma pieces taken from herbarium specimens (TNM F13635, TNM F13637). Both kinds of material were homogenized in liquid nitrogen. DNA was extracted with Plant Genomic DNA Extraction Miniprep Kit (Viogene, Taiwan), according to manufacturer’s protocol. Primer pair ITS1/ITS4 was used for amplification of internal transcribed spacer region, including ITS1, 5.8S, and ITS2, under PCR conditions, described in White et al. ([1990]). The DNA fragment of ribosomal large subunit gene (28S), was amplified with primers LR0R/LR5 (Moncalvo et al., [2000]), following PCR settings as described in Wu et al. ([2007]). Amplifications were run on a Mastercycler Gradient 5331 thermal cycler (Eppendorf, Germany). Amplification products were purified with a PCR-M Clean Up kit (Viogene) and sequenced with an ABI PRISM BigDye Terminator Cycle Sequencing Ready Reaction kit on ABI 3730 DNA sequencer (Applied Biosystems, USA). The resulting sequences were deposited in NCBI GenBank (Table 1).

**Table 1:** **Taxa used in this study, along with their specimen /strain numbers, locality information and GenBank accession numbers**

Species name	Isolate / Specimen voucher	Country of origin	GenBank accession no. for nrDNA	
			5.8S-ITS2	28S
*Hyphoderma cremeoalbum*	/ NH 11538 (GB)	Turkey	DQ677492	DQ677492
*Hyphoderma definitum*	/ GEL 2898		–	AJ406509
*Hyphoderma definitum*	/ FCUG 2426	Russia (Krasnodar krai)	AJ534293	–
*Hyphoderma definitum*	/ NH 12266 (GB)	Russia	DQ677493	DQ677493
*Hyphoderma granuliferum*	/ KHL 12561 (O)	Costa Rica	JN710545	JN710545
*Hyphoderma incrustatum*	KHL 6685 /		–	AY586668
*Hyphoderma litschaueri*	/ NH 7603 (GB)	Canada	DQ677496	DQ677496
*Hyphoderma litschaueri*	/ CFMR:DLL2011-050	USA	KJ140573	–
*Hyphoderma macaronesicum*	E09/57-9 / TFC:Mic.15981	Canary Islands	HE577027	–
*Hyphoderma medioburiense*	/ NH 10950 (GB)	Spain	DQ677497	DQ677497
*Hyphoderma nemorale*	EM 2793 /		**–**	AY586669
*Hyphoderma nudicephalum*	/ TMIC 33708	Japan	AJ534264	**–**
*Hyphoderma nudicephalum*	**/** TMIC 50048	Japan	AJ534265	**–**
*Hyphoderma nudicephalum*	**/** FCUG 2949	Japan	AJ534266	**–**
*Hyphoderma nudicephalum*	/ TMIC 30479	Japan	AJ534267	**–**
*Hyphoderma nudicephalum*	Wu 9508–225 /	China	AJ534268	**–**
*Hyphoderma nudicephalum*	Wu 9307–29 /	Taiwan	AJ534269	**–**
*Hyphoderma nudicephalum*	/ TMIC 50049	Japan	AJ534270	**–**
*Hyphoderma nudicephalum*	/ GEL 4727		–	AJ406510
*Hyphoderma obtusiforme*	/ KHL 1464		JN572909	–
*Hyphoderma obtusiforme*	/ KHL 11105		JN572910	–
*Hyphoderma obtusum*	JS 17804 /		–	AY586670
*Hyphoderma occidentale*	KHL 8469G /		–	AY586674
*Hyphoderma occidentale*	/ KHL 8477 (GB)	Sweden	DQ677499	DQ677499
Hyphoderma pinicola*	**Wu 0108–30 / TNM F13635**	**China**	**KJ885179**	**KJ885180**
Hyphoderma pinicola*	**Wu 0108–32 / TNM F13637**	**China**	**KJ885181**	**KJ885182**
Hyphoderma pinicola*	**Wu 0108–36 / TNM F13643**	**China**	**KC928278**	**KC928279**
*Hyphoderma prosopidis*	E09/58-9 / ARIZ:H.H. Burdsall 8479	USA	HE577029	–
*Hyphoderma roseocremeum*	NH 10545 /		**-**	AY586672
*Hyphoderma setigerum*	/ GEL4001		**–**	AJ406511
*Hyphoderma setigerum*	/ FCUG 1688	Finland	AJ534272	**-**
*Hyphoderma setigerum*	/ FCUG 1200	Norway	AJ534273	**-**
*Hyphoderma setigerum*	KHL 8544 /		-	AY586673
*Hyphoderma setigerum*	FCUG 1264 / NH 8544 (GB)	Sweden	FN907905	FN907905
*Hyphoderma setigerum*	FCUG 2499 /	Argentina	GQ409515	**-**
*Hyphoderma setigerum*	FCUG 2530 /	Argentina	GQ409516	**-**
*Hyphoderma setigerum*	FCUG 3038 /	South Africa	GQ409517	**-**
*Hyphoderma setigerum*	FCUG 3037 /	South Africa	GQ409518	**-**
*Hyphoderma setigerum*	CFMR FP101976 /	USA	GQ409519	**-**
*Hyphoderma setigerum*	CFMR HHB9443 /	USA	GQ409520	**-**
*Hyphoderma subtestaceum*	CFMR HHB11620 /	USA	GQ409521	-
*Hyphoderma subtestaceum*	CFMR MJL1536 /	USA	GQ409522	-
*Hyphoderma subsetigerum*	Wu 9508-155 /	China	AJ534275	**-**
*Hyphoderma subsetigerum*	/ TMIC 33552	Japan	AJ534276	**-**
*Hyphoderma subsetigerum*	Wu 9304-18 /	Taiwan	AJ534277	**-**
*Hyphoderma subsetigerum*	Wu 9202-15 /	Taiwan	AJ534278	**-**
*Hyphoderma transiens*	/ NH 12304 (GB)	Sweden	DQ677504	DQ677504
Mutatoderma heterocystidium**	/ NH 7574 (GB)	Canada	DQ677495	DQ677495
*Mutatoderma mutatum*	/ NH 12026 (GB)	Russia	DQ677498	DQ677498
*Phanerochaete sordida*	/ KHL 12054 (GB)	Norway	EU118653	EU118653

*Data in bold indicate the sequences obtained in this study.

**In GenBank under the name *Hyphoderma heterocystidiatum* (Burt) Donk.

### Sequence alignment and reconstruction of phylogeny

The datasets were composed of the sequences obtained in this study and taken from GenBank (Table 1), with the aim to elucidate phylogenetic distances between the new taxon and other *Hyphoderma* species. Two species of *Mutatoderma*, earlier known under *Hyphoderma*, were added to both 5.8S-ITS2 and 28S datasets. From about 50 ITS sequences of *H. setigerum* available in GenBank, we selected those representing the main clades within this species complex (Nilsson et al., [2003]). The selected taxa in the ingroup belong to residual polyporoid clade of Agaricomycetes (Binder et al., [2013]). *Phanerochaete sordida* (P. Karst.) J. Erikss. & Ryvarden, a member of phlebioid clade, was selected as an outgroup in both datasets.

Sequences were aligned on-line in MAFFT v. 7 (http://mafft.cbrc.jp/alignment/server), using E-INS-i strategy for ITS and G-INS-i for 28S (Katoh et al., [2009]). Before alignment, ITS1 and 28S segments were cut from the sequences where needed. Sequences that were too short were removed from the datasets after preliminary alignments. Final datasets were edited manually in MEGA v. 3.1 (Kumar et al., [2004]). Ready data matrices, together with resultant phylograms, were deposited in TreeBase (http://purl.org/phylo/treebase/phylows/study/TB2:S16046). The best-fit models of nucleotide evolution were estimated by MrModeltest v. 2.3 (Nylander, [2004]), with Akaike Information Criterion as a relative quality measure of the model (Posada and Buckley, [2004]). The input file for MrModeltest was generated in PAUP* v. 4.0b10 (Swofford, [2002]).

Bayesian analysis of phylogeny was performed in MrBayes v. 3.2.1 (Ronquist and Huelsenbeck, [2003]). Both 5.8S-ITS2 and 28S datasets were individually analyzed in two independent runs, each with four MC^3^ chains running for 1 million generations, with tree and parameter sampling every 500 generations. Burn-in was as default setting (discarding 25% of samples). The datamatrix of 5.8S-ITS2 was analyzed with different sets of parameters for two partitions, 5.8S and ITS2, according to best-fit models. FigTree v. 1.3.1 was used to view and capture the resultant phylograms and CorelDraw v. 9 for drawing the images.

## Results

### A key to species of Hyphoderma setigerum complex

1. Conspicuous capitate cystidia with naked, bulbous apices present, up to 14 μm wide apically, coarsely encrusted below the apex …………………………………… *H. nudicephalum*
2. Cystidia more or less cylindrical, seldom with naked capitate apex, but the apical bulb not exceeding 8 μm in width …………………………………………………………………………… 2
3. Basidia 2-sterigmate …………………………………………………………………………… 3
4. Basidia 4-sterigmate …………………………………………………………………………… 4
5. Basidiospores reaching 15–17.5 μm long, often suballantoid; basidia 25–32 μm long; septocystidia naked or weakly encrusted, often with thin (up to 0.5 μm) wall ………… *H. pinicola*
6. Basidiospores 10–12 μm long, only a little depressed adaxially; basidia 17–25 μm long; septocystidia heavily encrusted, typically with thick (up to 1 μm) wall …………… *H. bisetigerum*
7. Basidiospores 6–8 × 2.8–3.2 μm; known from Taiwan, Japan, China ……………………………………………………………………………… *H. subsetigerum*
8. Basidiospores 7–10(–14) × 3–5(–6) μm; worldwide ……………………………… *H. setigerum*

### Hyphoderma pinicola

**Figure 1:**
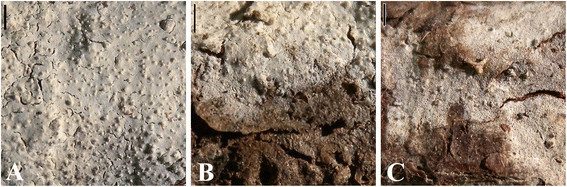
**Macromorphology of** ***Hyphoderma pinicola*** **(TNM F13635). A**, Basidioma in central part (holotype); **B**, Basidioma in marginal part (holotype); **C**, Thin, minutely porulose basidioma. Scale bars = 1 mm.

**Figure 2:**
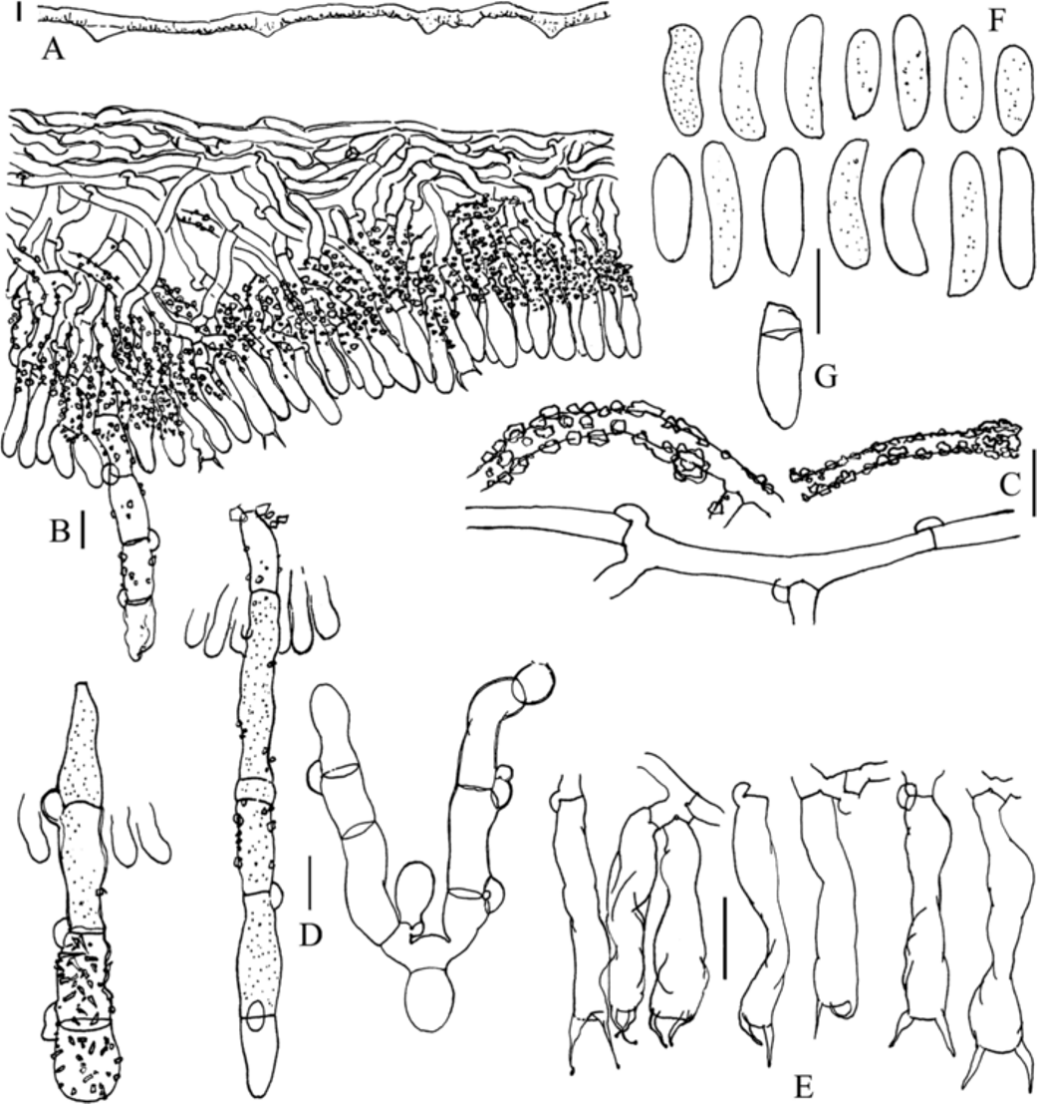
**Micromorphology of** ***Hyphoderma pinicola*** **(TNM F13637). A**, **B**, Vertical sections through basidioma; **C**, Naked and encrusted subicular hyphae; **D**, Two straight and one distorted septocystidia; **E**, Basidia; **F**, Normal basidiospores; **G**, Basidiospore with a false septum. Scale bars: for A = 100 μm; for B–G = 10 μm.

Yurchenko & Sheng H. Wu, sp. nov. Figures 1 and 2.

### MycoBank

804684

### Holotype

China. Yunnan Prov., Hoching County, Sungkuei, alt. 2200 m, on dead corticated branch of *Pinus yunnanensis*, coll. S.H. Wu & S.Z. Chen, 1 Aug 2001, *Wu 0108–32* (TNM F13637; isotype in MSK).

### Etymology

Specific epithet refers to the host preference (*Pinus*).

### Diagnosis

Basidioma thin; hymenial surface chalky white, warted; septocystidia scattered, almost naked and often thin-walled; basidia with two large sterigmata; basidiospores (10–)13–16(–17.5) μm long, predominantly suballantoid.

### Description

Basidiomata effused, membranaceous, 60–150 μm thick. Hymenial surface chalky white, minutely warted (3–4 warts/mm), between warts from the beginning minutely porulose, then continuous, minutely cracking with age. Margin zone up to 1.5 mm broad, concolorous with the main hymenial surface, abrupt or usually diffuse or slightly fibrillose. Hyphal system monomitic, all hyphae colorless, thin-walled, clamped at all primary septa. Subiculum thin, consisting of thin, fairly compact layer of more or less horizontal hyphae next to the substratum, and loose intermediate layer composed of variously oriented hyphae. Subicular hyphae moderately branched, 3–4.5 μm diam, naked to richly encrusted. Subhymenium not distinctly differentiated from subiculum. Subhymenial hyphae moderately to richly branched, 2.5–3 μm diam, moderately to richly encrusted (most incrustation dissolving in KOH solution). Cystidia of two kinds: (1) septocystidia scattered, projecting, irregularly cylindrical, straight to strongly twisted, simple or branched, with (2–)4–6(–8) and more predominantly clamped septa, apically near cylindrical or subcapitate, 65–180 μm long, up to 7–11 μm broad in swellings and the least 3.5 μm broad in constrictions, colorless, moderately thin-walled (walls up to 0.5 μm thick) to seldom thick-walled (up to 2 μm thick), naked or slightly encrusted, especially in basal part; (2) aseptate cystidia may be found in younger portions, slightly protruding, subcylindrical, 35–45 × (4.5–)6–7 μm, thin-walled, naked. Basidia subcylindrical or narrowly utriform, 25–28(–32) × 5–6.5 μm, colorless, thin-walled, naked, with two sterigmata measuring 6.5–7.5(–9) × 1–1.5(–1.8) μm. Spores cylindrical to allantoid, rarely slightly sigmoid, (10–)13–16(–17.5) × (3.5–)4–4.5(–6) μm (rarely up to 19.5 μm long), colorless, thin-walled, smooth, negative in Melzer’s reagent, acyanophilous, with a short rounded apiculus.

### Additional specimens examined

CHINA. Yunnan Prov., Hoching County, Sungkuei, alt. 2200 m, on dead twig of *Pinus yunnanensis*, coll. S.H. Wu & S.Z. Chen, 1 Aug 2001, *Wu 0108–30* (TNM F13635); Chuhsiung, Tzuhsishan, alt. 2400 m, on dead twig of *Pinus* sp., coll. S.H. Wu & S.Z. Chen, 2 Aug 2001, *Wu 0108–36* (TNM F13643).

### Distribution

The species is so far known only from the temperate belt in northwest part of Yunnan Province, China.

### Remarks

Another bi-sterigmate species from the *H. setigerum* complex, *H. bisetigerum* Boidin & Gilles, was described from Madagascar (Boidin and Gilles, [2003]). However, this species differs from *H. pinicola* in bearing shorter basidia, shorter basidiospores, and heavily encrusted septocystidia (see key). In the global survey of the *H. setigerum* complex (Nilsson et al., [2003]), the largest spores (12–14.5 × 4.5–5 μm) were found in the material from Greenland; these however are shorter than in *H. pinicola*. According to a detailed morphological study of *H. setigerum* in Belarus and northwest Russia (Yurchenko and Zmitrovich, [2001]), the largest spores do not exceed 14 μm long, with averages from 7.3 to 11.2 μm. The average spore size in *H. pinicola* specimens was 15.1 × 4.3 μm (holotype), 14.4 × 4.3 μm (TNM F13635), and 14.1 × 4.5 μm (TNM F13643). The preference of growing on coniferous wood is rare for *H. setigerum* s.l., and it is a distinctive ecological feature of *H. pinicola*.

### Molecular phylogeny

The 5.8S-ITS2 dataset analyzed by Bayesian analysis included 356 positions together with introduced gaps (99 positions in partial 5.8S, 86 of which were constant; 257 positions in complete ITS2, 66 of which were constant). MrModeltest suggested GTR + I + G as the best-fit model of nucleotide evolution for 5.8S + ITS2, K80 for partial 5.8S, and GTR + I + G for the whole dataset. The aligned datamatrix of partial 28S sequences included 882 positions, of which 820 were constant. The best-fit model of nucleotide evolution suggested for it by MrModeltest was GTR + I + G.

**Figure 3:**
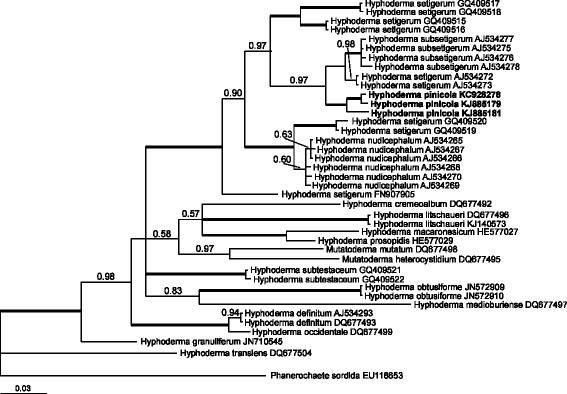
**Phylogram obtained to reveal the phylogenetic position of** ***Hyphoderma pinicola*** **via Bayesian analysis (5.8S-ITS2 dataset).** Numbers above branches denote Bayesian posterior probability (PP) value (if PP ≥ 0.50). Thick branches have PP ≥ 0.99. Scale bar for branch length indicates the number of nucleotide substitutions per site.

**Figure 4:**
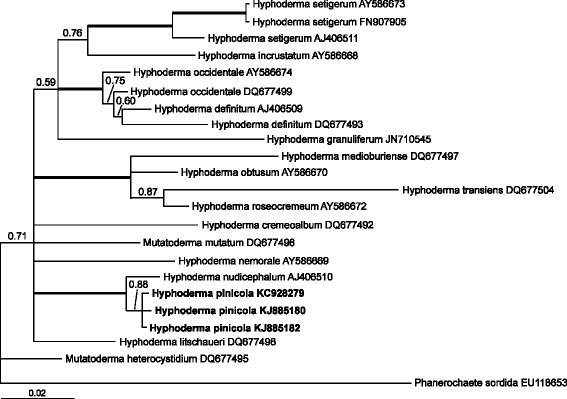
**Phylogram obtained to reveal the phylogenetic position of** ***Hyphoderma pinicola*** **via Bayesian analysis (28S dataset).** Numbers above branches denote Bayesian posterior probability (PP) value (if PP ≥ 0.50). Thick branches have PP ≥ 0.99. Scale bar for branch length indicates the number of nucleotide substitutions per site.

Both phylograms generated using Bayesian approach (Figures 3 and 4) confirmed that *H. pinicola* belongs to the genus *Hyphoderma*. According to the phylogram based on 5.8S-ITS2 (Figure 3), three specimens of *H. pinicola* constitute a well-supported clade (with Bayesian posterior probability value, PP = 1.00) within the *H. setigerum* complex. The phylogram based on partial 28S (Figure 4) demonstrates that three specimens of *H. pinicola* also constitute a separate clade (PP = 0.88). They belong to a highly supported clade (PP = 1.00) together with *H. nudicephalum* Gilb. & M. Blackw. A high degree of molecular divergence in ITS and 28S sequences supports specific status of *H. pinicola*.

## Discussion

A test of the application of molecular phylogeny in taxonomy is whether there is consistency between classification schemes based on molecular characters and morphological traits. Our results demonstrate certain congruence between morphological study and ribosomal gene sequence analyses. The phylogram inferred from 5.8S-ITS2 shows *H. pinicola* as a member of *H. setigerum* complex. All specimens of the *H. setigerum* complex constitute a separate, strongly supported clade. However, the *H. setigerum* complex appeared to be not monophyletic according to 28S-based phylogram. This phylogram shows that *H. pinicola* and *H. nudicephalum* are separate from *H. setigerum*. Basal branching order in 28S-based phylogram is not strongly supported by Bayesian posterior probability values, indicating that phylogenetic differentiation within the genus is not deep.

The previous comprehensive phylogenetic study of the *H. setigerum* complex (Nilson et al., [2003]) provided molecular grounds for recognition of *H. nudicephalum* and *H. subsetigerum* Sheng H. Wu. These species were defined morphologically: by characteristic cystidia in *H. nudicephalum* (Gilbertson and Blackwell, [1988]) and small spores in *H. subsetigerum* (Wu, [1997]). However, more than ten clades and subgroups were recognized and considered at species rank within this complex. As Nilsson et al. ([2003]: 651) noted, *H. subsetigerum* known from Asia (subgroup 7B) and *H. setigerum* s.s. from Northern Europe (subgroup 7B, AJ534272, AJ534273) constitute two different species because of incompatibility, geographic isolation and differences in spore size. *Hyphoderma pinicola* constitutes the third species in this small clade, possessing distinctive features in morphology, host preference, and distribution. The clade composed of long-spored specimens from Greenland is not only geographically, but also phylogenetically far from `*H. subsetigerum*’ clade (see Nilsson et al., [2003], Figure 1). This analysis revealed that the sister group to the `*H. subsetigerum*’ clade (i.e. the assemblage of *H. pinicola*, *H. setigerum* s.s., and *H. subsetigerum*) includes specimens of *H. setigerum* s.l. from Argentina (GQ409515, GQ409516) and South Africa (GQ409517, GQ409518). Both of these clades can be recognized as two independent species. Thus, after phylogenetic reconstructions, presumably new species from geographically separated areas appeared to be discriminated in highly supported clades on the basis of the 5.8S-ITS2 dataset only.

Two specimens of *H. subtestaceum* (Litsch.) Donk (GQ409521, GQ409522) were included in our phylogenetic study. According to *Mycobank*, *H. subtestaceum* is a synonym of *H. setigerum*. However, in the phylogram based on 5.8S-ITS2 sequences (Figure 3), these two specimens constitute a highly supported clade (PP = 1.00) separate from the clade that includes the *H. setigerum* complex. These results suggest that specimens from North America, called *H. subtestaceum*, belong to an independent species.

## Conclusion

*Hyphoderma pinicola*, known from Yunnan Province, China, and collected on dead wood of *Pinus yunnanensis*, represents the fifth named species of the *H. setigerum* complex.

## Authors’ original submitted files for images

Below are the links to the authors’ original submitted files for images. Authors’ original file for figure 1 Authors’ original file for figure 2 Authors’ original file for figure 3 Authors’ original file for figure 4
