# Supplementary figures and images for: Hyphoderma pinicola sp. nov. of H. setigerum complex (Basidiomycota) from Yunnan, China
Source: Bot Stud. 2014 Oct 9;55:71. doi: 10.1186/s40529-014-0071-5 (PMC5430344; doi:10.1186/s40529-014-0071-5)

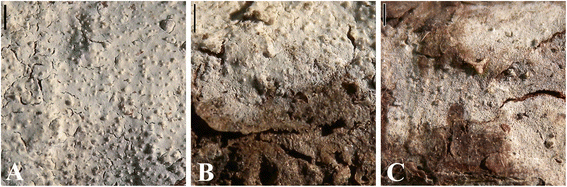

Supplement: Supplementary file 1 — Authors’ original file for figure 1 [file 40529_2014_9071_MOESM1_ESM.gif]

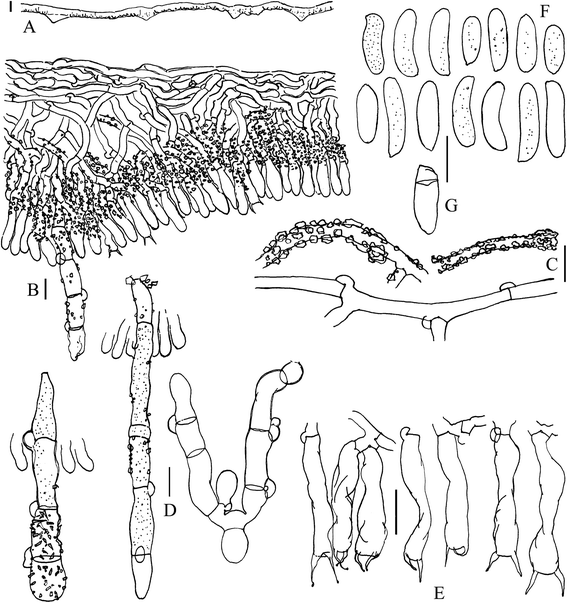

Supplement: Supplementary file 2 — Authors’ original file for figure 2 [file 40529_2014_9071_MOESM2_ESM.gif]

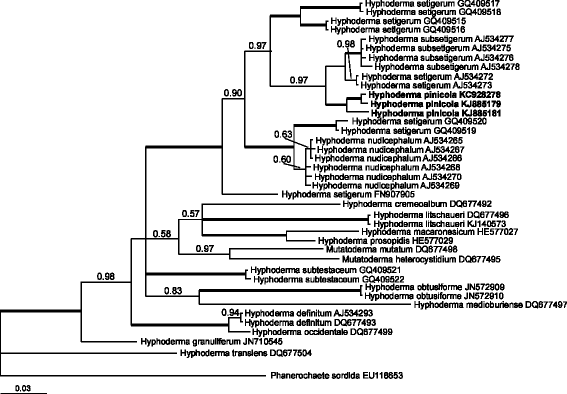

Supplement: Supplementary file 3 — Authors’ original file for figure 3 [file 40529_2014_9071_MOESM3_ESM.gif]

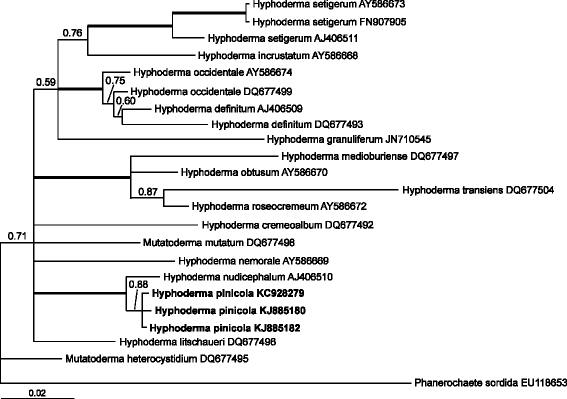

Supplement: Supplementary file 4 — Authors’ original file for figure 4 [file 40529_2014_9071_MOESM4_ESM.gif]
